# A Review of the Impact of Neuter Status on Expression of Inherited Conditions in Dogs

**DOI:** 10.3389/fvets.2019.00397

**Published:** 2019-11-13

**Authors:** Anita M. Oberbauer, Janelle M. Belanger, Thomas R. Famula

**Affiliations:** Department of Animal Science, University of California, Davis, Davis, CA, United States

**Keywords:** neuter, dog, spay, inherited disease, immune disease

## Abstract

Gonadectomy is an important reproductive management tool employed in many countries, and is highly prevalent in the US with an estimated 85% of dogs being neutered. Despite the societal benefits in pet population control, negative associations between neuter status, and health conditions have been reported in recent years. Most particularly observed are the consequences of early age neutering. Knowing that different physiological systems rely upon gonadal steroids during development and physiological maintenance, studies have been undertaken to assess the impact of neuter status on multiple body and organ systems. For some inherited conditions, neutering is associated with an increased risk of expression. Neutering has also been associated with altered metabolism and a predisposition for weight gain in dogs, which may confound the detected risk association between neutering and disease expression. This review summarizes the effects of neutering on cancer, orthopedic, and immune disorders in the dog and also explores the potentially exacerbating factor of body weight.

## Introduction

Gonadectomy, or more generally referred to as “neutering,” is a common procedure in many countries as a means to control reproduction in companion dogs and promoted extensively as a means to reduce dogs relinquished to shelters (1). It is also employed to alter undesirable behavioral characteristics such as aggression and roaming (2–4) although within the literature are disparate reports of aggressive dog behavior being greater among neutered dogs of both sexes (5–9) whereas other literature suggests that neutering decreases aggressive behavior (10, 11). Neutering has been associated with reduced relinquishment (12) and increased lifespans (13, 14). Additional advantages of neutering are the reduced incidence of reproductive disorders (15–18) and elimination of the behavioral and physical changes that occur during estrus in the female. However, with respect to neutering for convenience, ethical considerations have been raised (19, 20).

With neutering of dogs widespread within the United States, much published literature has been devoted to ascertaining the optimal age of neutering and assessing any health consequences associated with early or late neutering (21). For example, a report in 2004 evaluated dogs neutered at early or later ages and the authors found an association between health conditions such as hip dysplasia, urinary incontinence, and cystitis with early neutering (22). Such findings resulted in a greater scrutiny of the potential adverse effects of neutering on health conditions, especially since it is well-known that different physiological systems and behavioral responses rely upon gonadal steroids during development. Beyond the timing of neutering, research has targeted the impact of neuter status on physiology (23). In particular, cancer and orthopedic conditions have been evaluated because of the known association of gonadal steroid hormones on normal development in the case of the latter (24, 25) and on cancer progression in the case of the former (15, 26, 27). Recent studies have looked at the potential effects of neutering on multiple organ systems (28, 29).

The majority of studies assessing the impact of neutering are retrospective, and look at correlations between conditions and the neuter status of a dog. Retrospective studies only reveal associations some of which may be spurious. For example, in early onset conditions it is possible that neutering may have been a consequence of disease diagnosis rather than causal of the disease or the disease itself may have precluded neuter surgery; such nuances will not be revealed in a retrospective study. Nevertheless, retrospective studies can be instructive and reveal meaningful associations. Together the reports present a complicated picture of the health impacts of neutering and reinforce the need for a thorough consultation between veterinarians and their clients when considering elective neuter surgery. This review will focus on the risk associated with neutering for diseases considered to be heritable.

## Background

For diseases that involve the reproductive system, such as mammary, or testicular cancer, neutering has a profound effect on reducing and/or eliminating the incidence. For other disorders, there have been few reports investigating the relationship of neutering and disease expression, and all have been retrospective epidemiological studies. Studies that have included neuter status as an explanatory variable for disease expression often have concluded that neuter status was not associated with disease diagnosis or that being neutered was associated with improved health of the dog. Evaluating the studies together suggest that the association of neuter status and expression of many inherited disorders is minimal or equivocal across published studies, although as will be discussed below, for cancer, orthopedic, and immune-mediated diseases, neuter status has frequently been associated with increased risk. Inherited diseases of physiological systems that do not rely upon gonadal steroids appear to not be influenced by neuter status in the expression of those diseases.

## Epilepsy, Gastric Volvulus, and Eye Disorders

Idiopathic epilepsy has been reported to have no association (30, 31) or significant but modest positive associations (29, 32) across all breeds. In contrast, another all breed study reported a reduction of cluster seizures with neutering (33) although another study reported the exact opposite (34). Several breed specific studies did not report an association between neutering and idiopathic epilepsy although the data in some suggest that neutering might have a role in improving welfare by decreasing seizure frequency, duration, or euthanasia (35–38). The conflicting reports in the published literature have led researchers to conclude that no definitive sex or neuter effect can be determined at this time (39). Consistent with this view is that although sex steroids have been implicated in modulating seizure thresholds, their role in epilepsy remains unclear (40).

Similarly, being neutered has not been reported to alter the risk for canine gastric volvulus (41, 42) though in some reports, neutering is associated with reduced risk (29, 43, 44). The disagreement of whether neutering has an impact on gastric volvulus needs further, prospective exploration. In contrast, for inherited eye disorders, neutering has been reported to have no significant impact on expression though there are typically higher numbers of cases seen in neutered animals reflecting a general higher representation of neutered animals in the study population (29, 45, 46).

## Cancer

For cancers having an inherited component, there is a generalized trend for an increase in risk associated with neutering across breeds and sexes. The physiological mechanism underlying that breed specific susceptibility for cancer is unknown although it may be related to breed-specific genetic signatures of the major histocompatibility complex (47, 48), a component of the immune surveillance that can detect developing tumor cells (49) as well as other ancestry related susceptibility.

Health disorders seen among dog breeds often reflects their genetic heritage. For instance, the breeds of dogs selected for use in hunting and retrieving, the sporting breeds, cluster together in tightly related ancestral clades (50) suggesting that within breeds of those clades, there may be shared genetic susceptibility that underlies the association of risk for certain diseases. This risk may be enhanced with neutering. It is likely that the actual genetic susceptibility may differ between the breed groupings because, for example, the sporting group breeds are genetically distinct from the working group breeds. However, in some cases, though breeds may be unrelated, they are phenotypically similar due to the genetic selection employed to meet breed standards. Convergent selection may have resulted in similar biological pathways being altered in unrelated breeds that predisposes to common diseases. The risk observed in mixed breeds may reflect the amalgamation of diverse genetic susceptibilities from many different breeds.

For many cancers exhibiting a genetic predisposition, being neutered increases risk (26, 27, 29, 51–53). In contrast, the incidence of cancers associated with the reproductive tract are reduced. For instance, the risk of mammary tumors, known to have a genetic component (54, 55), is greatly reduced with neutering (56). Neutering also reduces the risk of testicular cancer and perianal adenomas (57).

When aggregated data for all dogs across multiple breeds are analyzed, neutering increases the overall risk of hemangiosarcoma, lymphoma, mast cell tumors, and osteosarcoma in both sexes although females exhibit a greater risk when neutered than seen for neutered males across all these cancers (29). Another broadly based retrospective study reported an increased risk associated for lymphoma and mast cell tumors across both sexes and only increased risk for hemangiosarcoma in females and osteosarcoma in males associated with neutering (58). An increase in lymphoma risk associated with neutering was reported for a study across more than 1 million dogs of multiple breeds (59, 60) and a multibreed study in South Africa found that neutered males were at a lower risk for lymphoma than intact dogs but neutered females were at a greater risk (61). In contrast, Modiano et al. (62) did not find a neuter effect on lymphoma in more than 1,200 dogs of many different breeds. Another published study using a smaller population reported that when compared to intact counterparts, the risk of mast cell tumors was 4 times greater in neutered females and only 1.3 times greater in neutered males (63). Likewise, osteosarcoma and hemangiosarcoma exhibit an elevated risk associated with neutering that is greater in neutered females than neutered males (51, 52, 64). A similar sex effect has been reported in studies that evaluated specific breeds and cancers (53, 65, 66). On the whole, there appears to be a generalized risk associated with neutering and expression of these cancers although there are variabilities which may reflect breed predisposition and which breeds comprised the populations under study.

For lymphosarcoma, hemangiosarcoma, and mast cell tumors, certain breeds have a higher proportion of neutered dogs presenting with cancer. Such a finding strongly indicates a genetic basis underlying cancer susceptibility. When three popular breeds, the Labrador and Golden retriever and the German shepherd dog, were compared (65–67), the risk for lymphosarcoma, hemangiosarcoma, and mast cell tumors was increased with neutering in Golden retrievers, most particularly for females, but not for either Labrador retrievers or German shepherd dogs. Across breed differences in cancer risk associated with neutering were also revealed when the published data reported in the supplemental figures of Belanger et al., were explored (29). Specifically, there are interesting cancer trends when breeds are grouped on functionality.

The American Kennel Club (AKC) assorts the different registered dog breeds into categories representing functionality. The breed groupings also often reflect similar genetic backgrounds that can provide insight into shared genetic susceptibility for various traits. For example, the “working breed” group is comprised of large breed dogs bred for particular jobs to assist humans; pulling sleds, guarding, and water rescue. Many breeds within the working group are genetically similar (50). The herding group likewise consists of breeds with similar function and often common ancestral breed contributions. For breeds categorized as “sporting” or “working” by the AKC, the risk of lymphosarcoma, hemangiosarcoma, mast cell tumors, and osteosarcoma tends to be much greater in neutered females than neutered males. Similar trends were seen for mixed breeds and for pit bull type dogs (29). Yet for some breeds and some cancers, there is equivalent risk across the sexes or reduced risk associated with neutering (29). Osteosarcoma is more frequently diagnosed in large breed dogs presumably as a result of disruption of osteoblast maturation (64, 68). For example, within the herding group, both neutered males and females of German shepherd dogs and Australian shepherds are at elevated risk for osteosarcoma. Ru et al. (64) also reported an elevated risk for osterosarcoma in German Shepherd dogs as well as large and giant breeds corroborating the association in the working and sporting groups seen in the Belanger et al., study. Another study reported the risk in female Rottweilers was greatly elevated (risk reported as a one in four chance) if dogs were neutered early (26) although an effect of neutering on Rottweilers was not detected in a smaller study (69). Interestingly, for breeds noted as being predisposed to osteosarcoma [reviewed in (69)] neutering in females was associated with an increased risk across all predisposed breeds whereas in males, the effect of neutering was nominal. Neutered males for breeds within the hound group, such as Afghan Hound, Basset Hound, Bloodhound, and Borzoi, were at a reduced risk for osteosarcoma than their intact counterparts. In contrast, neutering of females from this breed grouping, and for breeds generally predisposed for osteosarcoma, increased the risk for this cancer.

The variability along with sex and breed specificity noted in the literature suggests caution is needed when evaluating the potential impact of neutering on cancer expression. For example, osteosarcoma is associated with rapid bone growth and estrogens play a role in normal osteoblast differentiation (70). The removal of estrogens through ovariectomy stimulates the production and therefore activity of osteoblasts (71) and this osteoblastic expansion could possibly account for the elevated risk reported in neutered females, especially in breeds selected for large size which typically experience rapid growth and highly active osteoblasts.

When considering the effect of neutering on cancers, the mechanism of action is likely to be complex involving many factors including breed ancestry (phylogenetic relationships) as noted above, developmental stage at time of neuter, and physiological interactions of gonadal steroids with other physiological systems. For example, low circulating 25-hydroxycholecalciferol, a molecule in the Vitamin D synthetic pathway, is also associated with elevated risk for many human cancers (72). Studies have shown that vitamin D plays a critical role in modulating immune function, including the regulation of proteins involved in apoptosis, cell proliferation, and immune surveillance (73), the latter being particularly relevant in cancer detection by the body. Importantly for this discussion, plasma concentrations of 25-hydroxycholecalciferol are positively associated with gonadal hormones in humans (74, 75) and in dogs (76), although for the latter, dietary intake may have confounded the observed association. When gonadal hormones are removed with neutering, circulating plasma 25-hydroxycholecalciferol may be reduced thereby increasing susceptibility to cancer development as is seen for human cancers [e.g., (77, 78)] and reported for dogs (79). Additional evidence of the direct association between plasma 25-hydroxycholecalciferol and gonadal hormones was seen in the small study by Weidner et al. (80). The authors reported that the 25-hydroxycholecalciferol status subsumed the impact of sex/neuter status as a factor in cancer prevalence; that is, the impact of sex/neuter status was reflected completely in the plasma 25-hydroxycholecalciferol levels which might be expected if gonadal hormones had a role in regulating that molecule.

Another potential mechanism underlying elevated cancer risk associated with neutering relates to components of the body's immune surveillance. Neutering of male cattle, rabbits, and guinea pigs (81), and both male and female mice (82, 83) causes an enlargement of the thymus gland and a decrease in thymocytes programmed with a suppressor or cytotoxic phenotype (83). Suppressor and cytotoxic T cells are important in eliminating cancer cells. Perhaps the removal of the gonadal steroids might disrupt the ability to recognize and destroy precancerous cells thus creating a physiological tolerance for the proliferation of cancer cells.

## Orthopedic Disorders

Given the interaction of gonadal steroids and normal musculoskeletal development (24, 84), it is unsurprising that neutering impacts bone elongation in the dog (23) and thus, inherited conditions related to bone maturation. In one large study across many dog breeds, neutered males were at risk for hip dysplasia and neutered females for cruciate ligament damage with dogs of large and giant breeds at the greatest risk (85).

Certain categories of breeds, such as working, herding, and sporting breeds, show greater susceptibility to orthopedic disorders in general; specifically, dogs having large stature or great substance are at greater risk for hip and elbow dysplasia (86, 87). Within those breed groupings, some breeds prone to the orthopedic disorders did not show elevated risk for disease with neutering. For example, neutering did not worsen a risk for hip dysplasia for German shepherd dogs or Rottweilers, and in some breeds (e.g., Labrador retriever, Old English sheepdog) neutering was associated with reduced risk (29, 67). However, for other large breed dogs known to be predisposed for hip and elbow dysplasia, neutering was associated with an increased risk (e.g., Newfoundlands, Saint Bernards, Samoyeds). Terriers and toy breeds are not prone to hip and elbow dysplasia and neutering did not change that low risk. Taken together, there does not appear to be generalizable trends across breeds.

Small breed dogs, while being prone to patella luxation (86), were not at increased risk as a consequence of neutering in a study based upon records from the United States (29). Breeds for which patella luxation is not typically associated though did show an increased risk associated with being neutered (29). In contrast, in a United Kingdom retrospective study, the risk of patellar luxation increased more than 2-fold in neutered small dogs for both sexes (88).

In an all breed analysis, neutered males had elevated risk for intervertebral disk disease (IVDD). Certain breed morphology was especially linked to an elevated risk when neuter status was evaluated (29). Dachshunds are particularly noted for IVDD diagnosis with being neutered described as a risk factor for the condition (89); a recently published retrospective analyses demonstrated a correlation between early neutering (<12 months of age) and risk of intervertebral disc herniation in dachshunds of both sexes (90). For ruptured anterior cruciate ligament, neutering was associated with risk across all breed groupings although the working and large breed dogs appear to be overrepresented (29, 91, 92).

For inherited orthopedic conditions, such as hip and elbow dysplasia, removing the gonadal hormones that regulate the duration of bone elongation would be expected to alter growth patterns and subsequent joint alignment exacerbating any preexisting propensity for disease. Interestingly, horses, another athletic quadruped in which the vast majority of males are neutered early in life, exhibit disproportionate bone growth (93) and yet, do not appear to exhibit orthopedic deficits. The risks of orthopedic disorders associated with neutering seen in dogs may be a reflection of different stages of development at birth with horses being precocial whereas dogs are altricial and therefore more reliant on postnatal hormones for musculoskeletal development. Alternatively, extensive genetic selection applied in breed formation of the dog may play a role especially as a greater risk associated with neutering appears to be breed and ancestral lineage specific in some orthopedic diseases.

Taken together, despite some variability, for dogs of breeds in which a susceptibility to a particular orthopedic disorder exists, neutering may worsen that risk. It is quite likely that any orthopedic risk associated with neutering reflects the age of neutering. Linear bone growth represents an interplay of gonadal hormones, pituitary hormones, and growth factors (94) with gonadal steroids playing a prominent role in growth plate closure (95). The removal of the sex hormones prior to initiation of growth plate closure would promote the continued proliferation of cells within the growth plate thus permitting bone elongation that exceeds the normal growth period. Growth cessation differs for different bones within the body and the closure and growth is tightly orchestrated to optimize joint configuration. Neutering prior to cessation of bone growth would impact bone elongation and joint formation. Large breed dogs have a longer growth period than smaller stature dogs and thus bone/joint articulation may be more susceptible to environmental and exogenous perturbations in the large breeds. Recommending neutering based upon a standardized age would not take into account the growth stage of a particular dog; an early age of neuter recommendation might impact larger breed dogs disproportionately. Current perspectives argue for more individualized decision making as to the optimal time to neuter (96).

## Immune-Mediated Disorders

Female dogs, as also seen in humans and mice, are more prone to immune-mediated disorders (97, 98) and neutering appears to exacerbate that risk. The risk of certain immune diseases is elevated with neutering in both males and females: atopic dermatitis, autoimmune hemolytic anemia, hypoadrenocorticism, hypothyroidism, immune-mediated thrombocytopenia, inflammatory bowel disease, and systemic lupus erythematosus (14, 28, 99–102). For dogs diagnosed with hypoadrenocorticism, the relative risk of death is 2-fold higher than that seen for other dogs and in a large retrospective study in Sweden, breed by sex interactions were detected with some breeds exhibiting a female predisposition although in other breeds, both sexes were equally affected (103).

Even though neutered males have a greater, albeit moderate, risk for atopic dermatitis, hypothyroidism, immune-mediated thrombocytopenia, and inflammatory bowel disease when compared to intact males, the elevated risk seen in neutered females is much greater than that seen for neutered males; for some conditions, such as hypothyroidism and immune-mediated thrombocytopenia, the risk is 3-fold greater in neutered females than the elevated risk in males (28). Some studies have reported that neutering in both sexes was associated with increased risk of hypothyroidism (99, 100) although in smaller studies no relationship between neuter status and hypothyroidism was recorded (104, 105). In a study of American Eskimo dogs, neutering, especially for females, was a significant factor in diabetes mellitus expression (106) whereas other studies found that neutered males were more at risk for diabetes mellitus across many different breeds and females were at no greater risk if neutered (107, 108) and another multibreed study found neutering in both males and females increased risk of diabetes mellitus (109). Across studies, neutering was consistently associated with elevated risk of diabetes mellitus although various authors have suggested that the male gonadal hormones may be protective and age of neutering may not have been accounted for sufficiently in the studies (108). In a recent review, Niaz et al., reported that neutering females at ~1 year of age, prevented the development of diabetes mellitus in the dog by removing the ovary and the uterus and thereby hormones that activate diabetes (110). There is need however, for caution in interpreting retrospective studies centering on diabetes: potential alterations of body weight as a consequence of neutering may confound the risk of diabetes being attributed to neutering.

A sex effect on risk for immune disorders related to neutering is consistent with the risk noted above for cancers as impaired immune function is also associated with cancer progression (111). Gonadal steroids exhibit differential effects on the immune system and are believed to account for the sex specific susceptibility to immune and autoimmune disorders (112). Estrogens, acting through their cognate receptors, are critical modulators of both innate, and adaptive immune function (113). Estrogens increase the production of immunoglobulins, thereby enhancing the humoral immune response (114) as exemplified by females exhibiting a greater antibody response to vaccines (115). Estrogens can enhance the production of autoantibodies (116, 117) and preferentially stimulate IgM production (97), the immunoglobulin form most predominant in autoimmune disease (118). Thus, immunoglobulin production induced by estrogen has been implicated in mediating autoimmune pathologies (119) especially as estrogens impair the body's process of removing autoreactive B cells (115). In contrast, testosterone reduces humoral immunity, reducing immunocompetence, as seen in birds (120), lizards (121), fish (122), mice (123), and humans (124). Within mice, the impact of gonadal steroids on immune response has been shown to be genetically linked: the sexes of different mouse strains exhibit differential immune and antibody response (123). An influence of genetic background on the relationship between gonadal steroids and immune response may play a role in breed and sex specific immune-mediated diseases seen in the dog.

An altered immune response by neutering could be permissive for destruction of self-tissue, by failing to remove autoreactive antibodies, as in the case of autoimmune disorders. Neutered male mice show a slight increase in autoreactive antibodies (125) and neutered female mice exhibit depressed immune function (126). As noted above, in general, females produce higher numbers of autoreactive antibodies than males (97). Neutering female mice prone to severe autoimmune disease greatly accelerates the disease process and increases the T cells that are permissive for autoantibody production (127) and female mice prone to systemic lupus erythematosus also increase autoantibody production when neutered (128). Although similar studies of autoantibody production in response to neutering have not been undertaken in the dog, the findings in mice and other species may offer insight into the mechanism for the observed elevated risk of immune conditions with neutering and potential approaches to mitigate that risk.

## Weight as a Co-Factor

Although there exists conflicting information as to whether neutering alters metabolism thereby causing weight gain in dogs (129), neutering is viewed as a common predisposing risk factor for obesity, most especially for cats but also for dogs (130–137). Neutering associated with increased body weight is speculated to reflect energetic metabolic changes (138), dysregulated feeding behavior (139), and the removal of estrogen, a known regulator of food intake in cats (140). Obesity is a risk factor for many disorders and that risk between disease and obesity is overrepresented in females (141). This suggests a role for gonadal steroids across many inherited diseases and an interaction with body weight. Many inherited diseases do in fact show greater risk with neutering although that risk does not appear associated with weight gain. The risk of obesity associated with neutering is influenced by breed and sex of the dog (142). In turn, obese dogs are at an increased risk for numerous health conditions including those of genetic origin such as ruptured cruciate ligament, hip dysplasia, and hypothyroidism (137, 141, 143). Some cancers are also associated with obesity and a study by Weeth et al., found that the association in dogs between obesity and cancer varied by cancer type (144). Two small studies reported an association between excess weight and diabetes mellitus (145, 146) however in those studies the body weight was owner-reported and may reflect preconceived biases because the body condition information was collected after the dog was diagnosed with diabetes.

To account for increased body weight as a potential aggravating factor in the correlation between neuter status and disease expression, we analyzed the published data for 31 inherited conditions from our previous studies (29, 147) by weight category for the different breeds. For each breed, the distribution of recorded body weights was simply divided into thirds forming tertiles of low, medium, and high weight categories. The odds ratio (OR) of disease risk in neutered animals relative to disease risk in intact animals was then calculated by sex and by weight tertile within breed using the methodology previously detailed (29). From the published data, only breeds with at least 75 observations and one case for a given disease were used. With the extensive number of comparisons, the analyses are presented as heat maps to aid in visualization of the association of weight, disease risk, and neuter status (Supplemental Figure 1). For the heat maps, five risk categories were generated: neutering strongly lowered disease risk (depicted as green), neutering moderately lowered disease risk (depicted as light green), neutering had no decisive impact on disease risk (depicted as blue), neutering moderately increased disease risk (depicted as peach), and neutering strongly increased disease risk (depicted as red); white squares indicate insufficient data for analysis. Inspection of the heat maps depicting the risk associated with neutering for different AKC breeds categorized by weight, revealed an absence of substantive impact of weight on the disease risk associated with neuter status. There was however a notable exception to this generalization: underweight, neutered male Newfoundland dogs were at greater risk for the majority of inherited conditions evaluated in that study. Also of interest, being within the upper tertile of body weight reduced the disease risk associated with neutering for females of most breeds.

Additionally, we were interested in assessing if, for a given breed and disease combination, there was a trend in disease risk as animals progressed from the low to high weight tertiles. Such a trend would also provide evidence of weight being an additional risk factor for disease. Specifically, we counted the number of instances in which a breed/disease combination increased in disease risk (or decreased in disease risk, or remained in the same disease risk group) when comparing the low weight tertile group to the medium weight tertile group. Similarly, we counted the change in risk when comparing a breed/disease combination in the medium weight tertile with that of the high weight tertile. As seen in Table 1, across body weights the majority of instances related to disease risk associated with neutering and weight across all dog breeds indicated that there was no decisive impact of weight on risk. That is, weight did not exacerbate nor ameliorate the risk associated with neutering for a particular disease. This finding corroborates a retrospective study report in which weight was not found to be a risk factor for osteosarcoma (64).

**Table 1:** The number of observations across all breeds and all diseases (29) in which weight affected the risk for disease in neutered animals across three evenly-spaced, within breed, weight groups.

	**Males**			**Females**		
	**Weight**					
	**Low**	**Medium**	**High**	**Low**	**Medium**	**High**
**Disease risk**						
Strongly lowers	50	86	103	98	126	146
Moderately lowers	42	81	134	110	190	245
No impact	1,954	1,916	1,836	1,885	1,807	1,751
Moderately increased	32	7	13	25	11	0
Strongly Increased	20	8	12	25	9	1

As for any retrospective approach, there are limitations to associating body weight with the disease risk associated with neutering in that the recorded body weight may or may not be temporally relevant to the original disease diagnosis. Despite this limitation, the data do not provide compelling evidence that any weight gain that may be associated with neutering increases the risk of inherited disease expression as a consequence of neutering. Therefore, the association of the risk of neutering on the expression of the conditions appears to be a direct effect of the neutering and not a secondary association due to altered weight metabolism.

## Summary

In many cases, neutering is promoted as the means to reduce the number of dogs euthanized in shelters, although one study (148) reported that “no clear results were found demonstrating the impact of total spay/neuter procedures on shelter intake.” With 85% of the dogs in the United States neutered (149) and yet ~3.3 million dogs enter a shelter annually in the United States (150), there are reasons beyond a failure to neuter that account for the number of dogs relinquished to shelters. With that backdrop, neutering is not the sole answer to reduce shelter euthanasia. However, there are definite advantages to neutering beyond preventing unwanted pregnancies including offering substantial convenience for owners and a reduction or elimination of some reproductive disorders.

When evaluating the risks of neutering, the economic impact should be part of the equation. The advantages and costs of surgical neutering should be weighed against potential welfare impacts on the dog. For instance neutering is a relatively low cost solution to unintended litters, reproductive disorders such as pyometra, dystocia, mammary tumors, testicular cancer, and perianal cancers. Should a dog be diagnosed with one of the aforementioned conditions, remediation can run thousands of US dollars. Studies summarized here demonstrate that neutering is associated with increased risk for some inherited diseases. Financial costs of treatments for hip surgery, diabetes control, cancer chemotherapy, and hormonal replacement vary by region, severity of the condition, and the duration or follow up but typically run thousands of dollars. Costs to repair a cranial cruciate ligament injury was estimated to range from $1,000 to $3,500 US dollars (151, 152). However, those individual costs must be balanced against the societal costs of pet relinquishment, abandonment, and overpopulation. A study in 2004 summarized the societal costs of dog overpopulation in the US including those from shelter management, animal control, dog bites, and vehicular accidents; the overall impact greatly exceeded $1.5 billion (153). A more recent study estimates $2.4 billion just on the shelter costs alone (154). Furthermore, it is important to assess the relative risk of a disorder associated with neutering and whether the elevated risk warrants undue concern. For example, a modest 1.3-fold increase in risk of a disorder being expressed may be statistically significant but not of substantial biological concern when contrasted with the risk of remaining intact [e.g., risk of testicular cancer having a prevalence exceeding 25% in the dog population (17)].

Reviewing multiple studies that have encompassed multiple breeds with respect to the effects of neutering on risk of disease expression may provide insight to the underlying susceptibility between neutering and a disorder. In the formation of breeds, specific phenotypic traits may be genetically linked to other genes that predispose some breeds to health disorders on their own or when selection practices lead to an exaggeration of those traits such as in the case of intervertebral disc disease and brachycephalic obstructive airway syndrome (155). Selection for phenotypic traits may have also inadvertently been permissive for the presence of deleterious alleles that may predispose certain breeds to diseases whose expression pathways interact with gonadal steroids, thereby making a breed more vulnerable to the expression of inherited disorders with neutering. This information may aid in decision making as to if, and when, to neuter an individual dog.

The studies reviewed here are retrospective studies. Despite the limitations of retrospective analyses with the limited numbers of disease cases, breed, and sex category, and most importantly, the timing of the neuter, the findings provide substantial information on the association of neutering with the development of genetic diseases. Breeds predisposed to a disorder may be more susceptible to the risks associated with neutering. The elevated risk reported in neutered dogs, most especially females, for many of the diseases underscores the need for deep consultation with animal care providers on timing of the neuter procedure and consideration of the potential positive and negative consequences that may be associated with the removal of gonadal steroids on overall health.

## Author Contributions

JB: data extraction and collation, interpretation, and edited manuscript. TF: informed data collection, determined and executed optimal design analysis, and edited the manuscript. AO: project conception, design, analysis, and manuscript preparation. All authors read and approved the final manuscript.

### Conflict of Interest

The authors declare that the research was conducted in the absence of any commercial or financial relationships that could be construed as a potential conflict of interest.

## Supplementary Material

Supplemental Figure 1 Heat maps of risk associated with neutering in males and females by dog breed, assembled into AKC breed groupings, by body weight tertiles for inherited disorders. Five risk categories generated: neutering strongly lowered disease risk (depicted as green), neutering moderately lowered disease risk (depicted as light green), neutering had no decisive impact on disease risk (depicted as blue), neutering moderately increased disease risk (depicted as peach), neutering strongly increased disease risk (depicted as red), and white squares indicate insufficient data for analysis. Supplemental Tables 1, 2 provide the codes for the breeds and diseases evaluated.

Supplemental Table 1 Breed codes.

Supplemental Table 2 Disease abbreviations.
