# Supplementary material for: A Review of the Impact of Neuter Status on Expression of Inherited Conditions in Dogs
Source: Front Vet Sci. 2019 Nov 13;6:397. doi: 10.3389/fvets.2019.00397 (PMC6863800; doi:10.3389/fvets.2019.00397)
Supplement: Supplemental Figure 1 — Heat maps of risk associated with neutering in males and females by dog breed, assembled into AKC breed groupings, by body weight tertiles for inherited disorders. Five risk categories generated: neutering strongly lowered disease risk (depicted as green), neutering moderately lowered disease risk (depicted as light green), neutering had no decisive impact on disease risk (depicted as blue), neutering moderately increased disease risk (depicted as peach), neutering strongly increased disease risk (depicted as red), and white squares indicate insufficient data for analysis. Supplemental Tables 1, 2 provide the codes for the breeds and diseases evaluated. [file Data_Sheet_1.PDF]

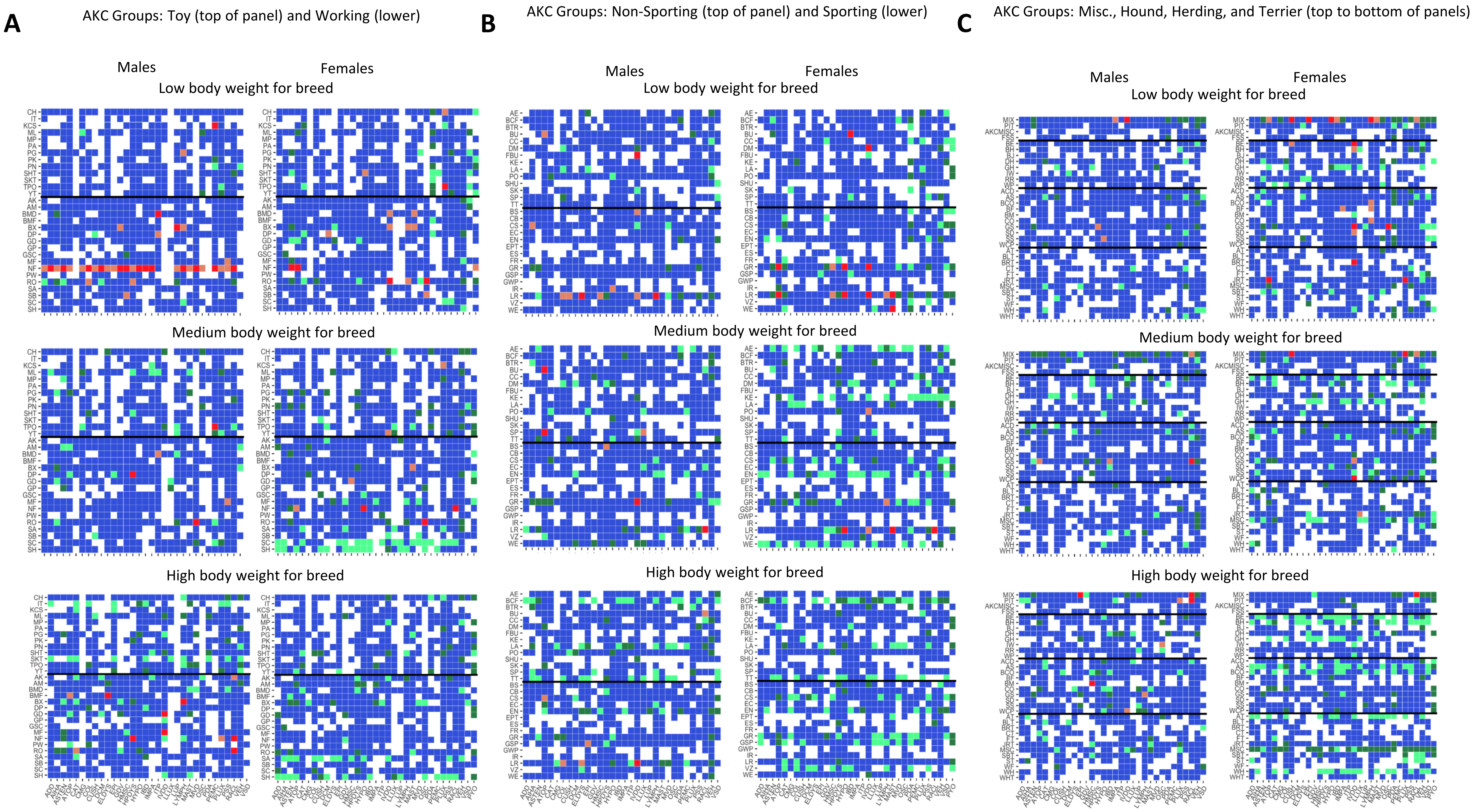
A
AKC Groups: Toy (top of panel) and Working (lower)
B
AKC Groups: Non-Sporting (top of panel) and Sporting (lower)
C
AKC Groups: Misc., Hound, Herding, and Terrier (top to bottom of panels)
Males
Females
Low body weight for breed
Medium body weight for breed
High body weight for breed
